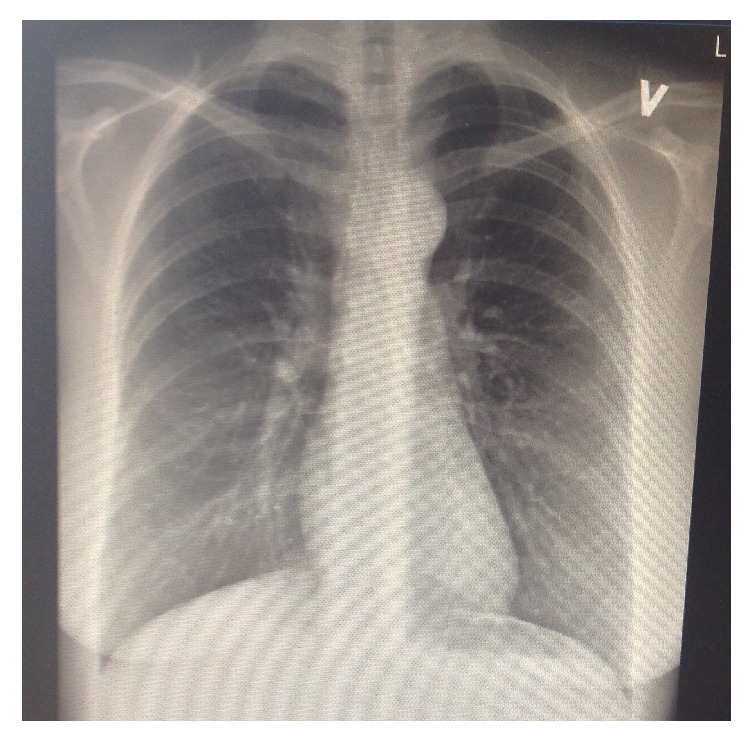# A Case with Severe Endometriosis, Ovarian Hyperstimulation Syndrome, and Isolated Unilateral Pleural Effusion after IVF

**DOI:** 10.1155/2017/8243204

**Published:** 2017-07-10

**Authors:** Negjyp Sopa, Elisabeth Clare Larsen, Anders Nyboe Andersen

**Affiliations:** The Fertility Clinic, Rigshospitalet, Copenhagen University Hospital, Copenhagen, Denmark

## Abstract

We present a very rare case of right-sided isolated pleural effusion in a patient with severe endometriosis who, in relation to in vitro fertilization (IVF), developed ovarian hyperstimulation syndrome (OHSS). Earlier laparotomy showed grade IV endometriosis including endometriotic implants of the diaphragm. The patient had no known risk factors for OHSS and only a moderate number of oocytes aspirated. She received, however, repeated hCG injections for luteal support. The patient did not achieve pregnancy but was hospitalized due to pain in the right side of the chest and dyspnoea. A chest computed tomography (CT) showed a pleural effusion on the right side. Total of 1000 ml of pleural fluid was drained after a single thoracentesis. After three days, the symptoms and fluid production ceased. Ascites is a common finding in OHSS, but pleural effusions are rare. Further, isolated pleural effusions have not previously been described in a patient with endometriosis. We suggest that the repeated hCG injections induced effusions from the endometriotic lesions at the diaphragm and as a consequence this patient developed isolated hydrothorax.

## 1. Case Report

**Figure 1:**
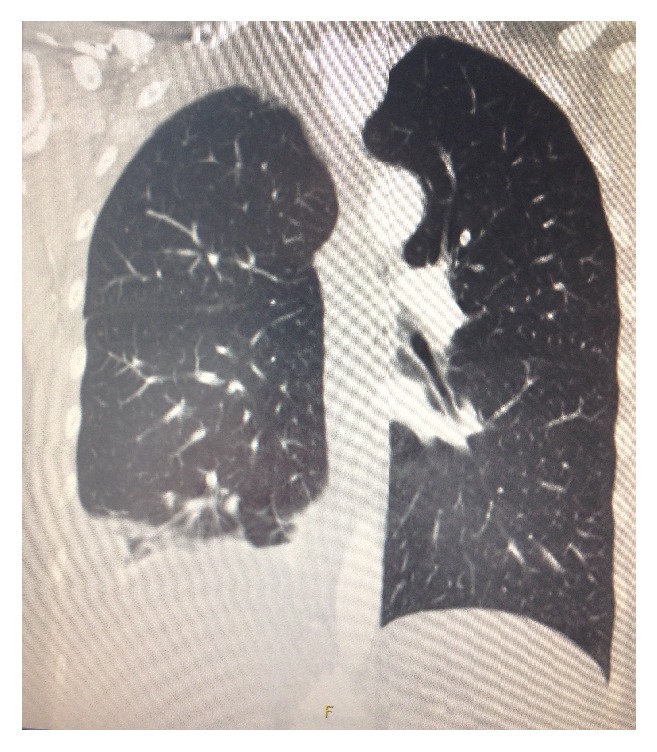


The case is a 29-year-old woman with a history of grade IV endometriosis and infertility. In 2011, she had surgery twice where endometrioses were diagnosed in the pelvic organs, the bowel, and the diaphragm. In 2015, she was referred to the Fertility Clinic after 1 year of infertility. Anti-Mullerian hormone (AMH) was 15 pmol/L. Initially, three intrauterine inseminations were done followed by IVF treatment with a standard antagonist protocol, where she received follitropin alpha (Gonal-f®) 150 IU daily for eight days. Choriongonadotropin (hCG; Ovitrelle) 6500 IU was given to induce ovulation. Nine oocytes were collected from eleven follicles. Two blastocysts were transferred and luteal support was given using vaginal progesterone. She did not become pregnant and did not develop OHSS. The second IVF treatment involved an antagonist protocol with Menotropin (hMG; Menopur®) 187.5 IU daily for 7 days. To induce ovulation she now received hCG (Pregnyl®) 10.000 IU. Five follicles were aspirated, and four oocytes were retrieved. On day 2, one embryo was transferred and she did not become pregnant. In the luteal phase, vaginal progesterone was supplemented with hCG 1500 IU every third day. Three doses were given. Thirteen days after the second oocyte retrieval, the patient was hospitalized primarily due to pain in the right side of the chest and dyspnoea. Initial examination by a cardiologist revealed tachypnea, dyspnoea, and tachycardia. Saturation was 100%, respiratory rate was 18/min, there was no rise in temperature, pulse was 82 beats/min, and blood pressure was 120/83 mmHg. There was no abdominal distension or ascites and no signs of thrombosis of the lower or upper extremities. Electrocardiogram (ECG) and blood tests were normal except for slightly elevated leukocytes 12.6 × 10^9^/l. On suspicion of pulmonary embolism, a computed tomography (CT) was made, showing a pleural effusion on the right side. There were no signs of pulmonary embolism (Figure 1).

The patient was then transferred to the gynecological department and admitted due to suspicion of OHSS. A pelvic ultrasound showed enlarged ovaries on both sides measuring 5,7 cm × 3,9 cm (right ovary) and 7,8 cm × 6,3 cm (left ovary) and a minimal amount of ascites. Thoracentesis was performed in local anesthesia by ultrasound guidance. A 7F pigtail catheter was introduced. Total 1.000 ml of clear yellow pleural fluid was drained during thoracentesis. Analysis of the sample showed negative cytological test for endometriotic cells and culture showed no signs of infection (bacteria and fungi). The patient received low molecular weight heparin Innohep® (Tinzaparin) 4500 IU subcutaneous prophylactically for 10 days.

After 3 days, the symptoms and fluid production ceased, and repeated chest X-ray showed no pleural effusion (Figure 2).

The patient recovered completely and was discharged.

## 2. Discussion

To our knowledge, this is the first case of OHSS with pleural effusion as the only clinical manifestation in a young woman undergoing IVF due to severe endometriosis. According to evidence-based Danish Clinical Guidelines, our case was classified as having a mild degree of OHSS since the largest ovarian measurement was less than eight cm [1, 2].

OHSS is a relatively common iatrogenic complication to controlled ovarian stimulation occurring in 0.5–5% of women undergoing IVF [3, 4]. The syndrome normally presents with ovarian enlargement and an acute fluid shift into extravascular spaces primarily causing accumulation of ascites in the abdomen. The pathogenesis is partly related to increased vascular permeability [5]. Risk factors are women with polycystic ovarian syndrome (PCOS), a high ovarian reserve, that is, elevated levels of anti-Mullerian hormone (AMH) and a high antral follicle count (AFC), previous OHSS, young age, high doses of rFSH, high number of oocytes collected, and high oestradiol levels at the end of stimulation [6]. Additionally luteal support using repetitive doses of hCG is known to increase the risk of OHSS, as reviewed by Fatemi et al. [7], and substantiated in the Cochrane Systematic Review including 94 randomised controlled trials comparing different luteal phase support regimens. The conclusion was that the use of repeated hCG injections as luteal support does increase the risk of OHSS [8].

Our case had none of the OHSS risk factors, except that she was exposed to repeated hCG injections. Further, in her first ART cycle, she had no OHSS or pulmonary complaints despite the fact that more oocytes were retrieved. She did however not receive repeated hCG injections in her first ART cycle.

HCG has multiple actions, including effects on the endometrium and endometrial angiogenesis during early implantation [9] Further, hCG stimulates corpus luteum to produce progesterone but also to some degree estrogen which is known to activate and maintain endometriosis. As such the endometriotic implants at the diaphragm may have been stimulated repeatedly by exogenous hCG and thus caused the effusion. Apposing this explanation is that her abdominal endometriosis did not cause effusions and thus ascites.

Severe OHSS is estimated to occur in 1% of women undergoing IVF [10, 11]. Symptoms include massive ascites, decreased effective blood volume, oliguria, thromboembolic complications, pleural and pericardial effusions, and sometimes even death. The occurrence of pleural effusion, that is, hydrothorax in patients with OHSS, is below 10% [10].

Endometrial implants, such as at the diaphragm, pleura, and pericardium, are uncommon. The cause of such implantation sites is unknown, but the theories include metaplastic transformation, lymphatic, hematogenous, and transdiaphragmatic migration [12, 13]. Thoracic endometriosis syndrome (TES) is a rare and complex disease, but nonetheless it is well described [13]. Symptoms include haemothorax, pneumothorax, and haemoptysis. TES in relation to IVF has been described in two case reports. In the first case, the patient presented with early OHSS three days after oocyte retrieval. A chest computed tomography showed bilateral pleural effusion and a subsequent thoracentesis revealed hemorrhagic pleural fluid. This case had 30 oocytes retrieved and developed OHSS with hemorrhagic pleural effusion as described above [14]. The second case developed pneumothorax after in vitro fertilization and embryo transfer. She had 13 oocytes collected [15].

Pleural effusion without intra-abdominal ascites is an extremely rare presentation of OHSS [16]. The first case of isolated pleural effusion associated with OHSS was described in 1975 [17]. Since then only few case reports have been published and none of these cases had endometriosis [17–20]. As described previously, pleural effusion mainly occurs in the severe forms of OHSS and in general together with other signs of OHSS. Predominantly, pleural effusion develops on the right side [21], the explanation being that lymphatic drainage may differ between the two sides and that the diaphragmatic recess is greater on the right side. It is also a possibility that pleural effusion is derived from a liquid shift from abdominal ascites [21, 22]. To our knowledge, there are no data explaining why pleural fluid does not drain into the abdominal cavity.

To conclude, we hypothesize that this patient, who had only 4 oocytes aspirated, developed isolated hydrothorax as a result of repeated hCG injections in the luteal phase. The theory behind this is that the exogenous hCG stimulated the endometriotic lesions at the diaphragm and as a consequence induced the pleural effusion. As the patient did not achieve pregnancy, her symptoms ceased when exogenous hCG was withdrawn.

## 3. Summary

We present a rare manifestation of OHSS, more specifically isolated pleural effusion. Furthermore, it developed in an endometriosis patient with a rather low response to controlled ovarian stimulation The patient was however given repetitive doses of hCG, which is known to increase the risk of OHSS. The pleural effusion may be caused by hCG induced stimulation of the endometriotic lesions on the diaphragm

## Figures and Tables

**Figure 2:**